# Date Palm Fruit (*Phoenix dactylifera*): Effects on Vascular Health and Future Research Directions

**DOI:** 10.3390/ijms22094665

**Published:** 2021-04-28

**Authors:** Yousef A. Al-Dashti, Roberta R. Holt, Carl L. Keen, Robert M. Hackman

**Affiliations:** 1 Department of Food and Nutrition Science, College of Health Sciences, Public Authority for Applied Education and Training, Shuwaikh 70654, Kuwait; 2 Department of Nutrition, University of California Davis, One Shields Avenue, Davis, CA 95616, USA; rrholt@ucdavis.edu (R.R.H.); clkeen@ucdavis.edu (C.L.K.); rmhackman@ucdavis.edu (R.M.H.); 3 Department of Internal Medicine, University of California Davis Medical Center, Sacramento, CA 95817, USA

**Keywords:** polyphenols, flavonoids, cardiovascular, inflammation, lipids, oxidative stress, endothelial function

## Abstract

Cardiovascular disease is a leading cause of death globally, presenting an immense public and economic burden. Studies on cardioprotective foods and their bioactive components are needed to address both personal and public health needs. Date fruit is rich in polyphenols, particularly flavonoids, certain micronutrients, and dietary fiber, which can impact vascular health, and have the potential to attenuate vascular disease in humans. Data from in vitro and animal studies report that consumption of date fruit or extracts can modulate select markers of vascular health, particularly plasma lipid levels including triglycerides and cholesterol, indices of oxidative stress and inflammation, but human data is scant. More investigation is needed to better characterize date polyphenols and unique bioactive compounds or fractions, establish safe and effective levels of intake, and delineate underlying mechanisms of action. Implementing scientific rigor in clinical trials and assessment of functional markers of vascular disease, such as flow-mediated dilation and peripheral arterial tonometry, along with gut microbiome profiles would provide useful information with respect to human health. Emerging data supports the notion that intake of date fruit and extracts can be a useful component of a healthy lifestyle for those seeking beneficial effects on vascular health.

## 1. Introduction

Lifestyle choices such as diet and physical activity can create risk factors for several chronic diseases including cardiovascular disease (CVD), diabetes, and certain cancers [1,2]. Worldwide, chronic diseases are projected to cause USD 17.3 trillion of cumulative economic loss between 2011 and 2030 due to increased healthcare expenditures, reduced productivity, and lost capital [3]. Prevention and risk-reduction strategies, including dietary recommendations, are crucial to stem this burden. In addition to guidelines on items to avoid, emphasis on health-promoting foods that complement current dietary strategies is key to the prevention and treatment of numerous chronic diseases [4].

Current dietary guidelines advocate beneficial patterns that share several key characteristics, including abundant intakes of fruits, vegetables, nuts and seeds, legumes, and whole grains, as well as seafood, yogurt, and vegetable oils, while minimizing the intake of red and processed (sodium/nitrate-preserved) meats, refined grains, starches, and added sugars [3,5]. Fruits and vegetables (e.g., citrus, berries, apples, cruciferous and green leafy vegetable sources) are rich in many essential nutrients and other bioactive compounds that can provide protection against many chronic diseases [6,7]. Dietary recommendations promote the consumption of at least five to nine servings of a variety of fruits and vegetables per day in a 2000 kcal diet [3,5], which provide abundant amounts of vitamins (e.g., ascorbic acid, folate, pro-vitamin A), minerals (e.g., potassium, calcium, and magnesium), fibers, and a diversity of bioactive phytochemicals such as polyphenols (e.g., flavonoids and phenolic acids) and carotenoids (e.g., carotenes and lycopene) [8]. An increased intake of polyphenols, particularly flavonoids, has been associated with a decreased risk for CVD [9,10] through improved endothelial function, and a reduction in platelet reactivity, low-density lipoprotein [LDL], and blood pressure [11,12,13,14].

Date palm fruit (*Phoenix dactylifera*), a species of the family Arecaceae that is rich in many essential nutrients and polyphenols, is one of the most commonly consumed fruits in the Middle East and North Africa [15]. Date palm fruit, which is termed simply as dates in this review, is cultivated throughout the Middle East and to an increasing degree in other regions of the world including parts of Central and South America, Europe, India, and the United States [16]. Consumer demand for dates continues to increase. The top countries produced about 3.5 million metric tons in 1990, around 6.5 million metric tons by 2000, and in excess of 7.5 million metric tons by 2014 [17].

Several biological activities, proposed mainly based on in vitro and animal models, have been described with respect to potential health effects of dates. These include support of oxidant defense [18,19], anti-inflammatory [20] and gastroprotective effects [21], and anticancer activity [15]. With the high incidence of CVD and diabetes worldwide, a comprehensive review of dates and their potential value in promoting vascular health is timely. Here, we focus on the roles of dates to affect markers of cardiovascular function, with particular attention to their beneficial actions in humans. Future research directions concerning dates are also suggested.

## 2. Historical Perspective

Date trees are among the oldest in the world, and are an important fruit crop in Middle Eastern countries [22,23]. Dates have significant religious importance for Muslims, where the fruit is mentioned in many sections of the Holy Quran for its nutritional and medicinal values [24]. This fruit has been used traditionally to break the fast during the holy month of Ramadan in Arabic and Islamic countries [25,26]. The earliest examples of the use of dates in the Middle East come from two sites, Sabiyah in Kuwait and the island of Dalma in the United Arab Emirates, as evidenced by carbonized date seeds and stones [24,27]. Dates have a special social status among Middle Eastern countries (e.g., Kuwait, Saudi Arabia, Bahrain) and with Arabs in general, as dates and date-based foods are served during most auspicious occasions and events, such as weddings, births, family gatherings, and religious holidays [28]. Although dates are admired for their nutritional and health-promoting properties by the natives of the Middle East and northern Africa, the fruit is less recognized in other regions of the world due in part to limited scientific documentation derived from Islamic prophetic traditions [29]. 

## 3. Cultivar and Composition

Many date cultivars are grown around the world that differ in size, taste, color and degree of ripeness when consumed [30]. The four main ripening stages are primarily known by their Arabic names (kimri: unripe; khalal: full-size, crunchy; rutab: ripe, soft; and tamar: final stage, ripe, reduced moisture) [31]. The chemical and functional composition of dates is significantly altered during the ripening process [32], with levels of sugars increasing and vitamin, mineral, and fiber levels gradually decreasing on a weight basis [33,34]. Ripening reduces the content of phenolic acids (i.e., hydroxybenzoic acids and hydroxycinnamic acids) and flavonoids (i.e., flavonoid glycosides, catechin flavanol, and anthocyanidins), as evidenced with Ajwa dates [35]. Polyphenol concentrations also vary by cultivar. For example, the khalal stage of the Ajwa cultivar contained significantly higher levels of the anthocyanidin petunidin (~31 mg/100 g) compared to Barni and Khalas cultivars at a similar stage of ripeness [35]. The phenolic content of Amari dates (phenolic acids: 4.27 μmol gallic acid equivalents (GAE)/g; flavonols: 1.37 μmol GAE/g) was also reported to be greater than the fraction isolated from Hallawi dates (phenolic acids: 0.38 μmol GAE/g; flavonols: 0.43 μmol GAE/g) at the same stage of ripeness [36]. The total phenolic content of different Iraqi dates also varied, ranging from 331 to 475 mg GAE/100 g, which are concentrations higher than other fruits such as apple, blueberry, orange, pomegranate, papaya, banana, and red grape [37,38]. In contrast, others have reported that the polyphenol content in the earlier stages of date ripening to be similar to that in apples, but lower than that in an extract of various citrus fruits [35]. Delineating the composition, variety, and ripening stage of dates and their bioactive fractions is important when designing and interpreting research studies. For consistent compositional reporting, standardization of extraction and analytical methods is needed.

Dates are relatively rich in kilocalories and contain a substantial percentage of carbohydrates (around 73% of the dry weight), which are predominately glucose (~90%), fructose, and sucrose [35,39]. The fruit also contains a significant amount of dietary fibers (estimated at 6.4 to 11.5 percent of the dry weight) including pectin, hemicellulose, lignin, resistant starch, and soluble fiber. Around 100 g of dates, equivalent to seven to nine fruits, provide 25–30 g of dietary fiber [40], which is 100% of the current US recommendations [41]. Date fruits contain protein (approximately 3%) and 23 different amino acids that are not commonly found in other fruits [39]. A variety of micronutrients are found in dates, including vitamins A, B-complex and C, and minerals such as calcium, magnesium, copper, sodium, phosphorus, zinc, selenium, fluorine, potassium, and iron [42]. Variability in the polyphenol content of dates exists, as well as in the macro- and micronutrient levels, depending on the cultivar and degree of ripeness, along with geographic location and environmental conditions [34,35].

## 4. Potential Benefits of Dates for Cardiovascular Health

Worldwide, CVD is the leading cause of death, taking an estimated 17.8 million lives in 2017, and is expected to account for more than 22.2 million deaths in 2030 [43]. An estimated 54% of deaths from noncommunicable disease in the eastern Mediterranean region are due to CVD and by 2030, an estimated 44% of the US population is projected to suffer from some form of CVD [44]. Age-standardized prevalence rates of CVD per 100,000 for both sexes are particularly high in North Africa and the Middle East, Central Asia and North America, ranging between about 7066 to greater than 9266 [45].

A number of risk factors are associated with the development and progression of CVD. While constitutional (non-modifiable) risk factors such as family history, age and sex cannot be controlled, lifestyle factors related to hypercholesterolemia, hypertension, hyperglycemia, obesity, physical inactivity, and smoking can be modified and can significantly impact cardiovascular health [44,46]. The presence of cardiac risk factors can be associated with vascular changes, and ultimately, the development of atherosclerosis, the underlying pathological process of CVD [47,48].

Atherosclerotic CVD is a chronic inflammatory disease and disorder of lipid metabolism, initiated by endothelial dysfunction and damage promoted by immune-related mechanisms that interact with platelets, leukocytes and low-density lipoprotein cholesterol (LDL-C) to initiate and propagate formation of lesions [49,50]. Vascular homeostasis is maintained, in part, by the vasodilators nitric oxide (NO), prostacyclin, endothelial derived hyperpolarizing factors, and vasoconstrictors such as thromboxane and endothelin-1(ET-1) [51,52]. These mediators also help regulate smooth muscle cell proliferation, inflammation and platelet activation [53,54]. In general, endothelial dysfunction occurs due to a disruption of the balance and regulatory function between vascular smooth muscle relaxing and contracting factors, growth promoting and inhibiting factors, and pro- and anti-atherogenic factors, characterized as a state of endothelial activation [55,56].

Diet and physical activity are essential components of a healthy lifestyle, which play important roles in the primary and secondary prevention of chronic diseases such as CVD [3,57]. Several bioactive dietary components are present in heart-healthy dietary patterns abundant in fruits, vegetables, nuts/seeds, and whole grains, including mono- and polyunsaturated fats, essential vitamins and minerals, phytochemicals such as polyphenols, and a variety of nondigestible carbohydrates (fibers and resistant starches) that either alone or through their interactive effects are thought to promote cardiovascular health [58,59]. Understanding how specific plant foods may be beneficial can provide further insight for future refinements of dietary and public health recommendations, especially since fruits and vegetables vary greatly in their profile of bioactive compounds.

### Effects of Date Palm Fruit

Most studies on the vascular-related effects of dates have focused on cholesterol and lipid regulation, and oxidant defense and inflammatory responses (Table 1). An in vitro study, using a colorimetric assay, demonstrated a Brazilian date fruit could inhibit angiotensin-converting enzyme activity [60], a potentially important target mediating blood pressure both in the pulmonary circulation and endothelial cells. Results from in vitro oxidant defense assays have been further supported by ex vivo and in vivo animal studies [61,62,63,64,65]. These studies demonstrated positive effects of different date cultivars (e.g., Hayani, Ajwa, Honey, Bam, Sahroon, Zahedi, and Kharak) against a variety of toxicants that produce free radicals, including carbon tetrachloride, isoproterenol (ISO), cadmium, and from the oxidant-generating streptozotocin-induced diabetic rat model. The protective effects of dates against oxidative stress were attributed to improved activities of oxidant defense enzymes such as catalase (CAT), superoxide dismutase (SOD), glutathione peroxidase, glutathione reductase, and glutathione S-transferase, along with a significant reduction in malondialdehyde. Moreover, dates were demonstrated to diminish oxidative damage, inflammation and apoptosis in cardiac tissue of ISO-treated rats [63]. Oral administration of lyophilized Ajwa date extract (250 and 500 mg/kg body weight) downregulated the expression of pro-inflammatory cytokines (interleukin [IL]-6, IL-10 and tumor necrosis factor-alpha [TNF-α]) and apoptotic markers (caspase-3 and Bax) in injured Wistar rat heart tissue, further supporting the anti-inflammatory and anti-apoptotic potential of dates against ISO-induced myocardial damage [63]. A more recent study investigated the possible cardioprotective effects of a nanopreparation mix of Ajwa date fruits and seeds on doxorubicin (DOX)-associated cardiotoxicity in Wistar rats by studying hemodynamic, electrocardiological, and biochemical changes [66]. The results obtained suggest that rats pre-fed 1.4 g/kg of the preparation one hour before DOX infusion were protected from a significant elevation in left ventricular pressure observed in the control group. In addition, pretreatment with the preparation prevented DOX-associated ischemia and increased the antioxidant capacity of reduced glutathione in cardiac tissue, compared with the untreated group. Quantification of Ajwa pits and a mixture of Ajwa fruit/pits reported that the pits contained 47.4 g/kg of total flavonoids, while the mix of pits with fruits contained 23.8 g/kg of total flavonoids, including epicatechin-phloroglucinol and epicatechin.

Extracts of high concentrations of phenolic and flavonoid compounds in four different varieties of dates (Berhi, Khalase, Khenizi, and Reziz) exerted favorable effects in protecting and repairing tissue injury following myocardial infarction (MI) induced either by ISO or temporary ligation of the left anterior descending coronary artery in a rodent model via oxidant defense activities and mobilization of circulating progenitor cells from bone marrow and peripheral circulation [71]. In this regard, oral pretreatment with date extracts for a period of 28 days prior to ISO injection significantly improved the state of MI compared to the control group. Elevated levels of glutathione, SOD, and CAT, and reduced levels of thiobarbituric acid reactive substances (TBARS) in heart tissue of rats were noted [71]. A dose-dependent effect was evident in which 400 mg/kg had significantly greater effects compared to 200 mg/kg in most cases. Interestingly, following MI induction, compared to controls, date extracts significantly increased circulating levels of CD34 and CD133 positive progenitor cells that are involved in tissue repair. The quantity of total phenolics in the dry plant material varied from 21.53 ± 0.90 to 26.82 ± 0.92 mg GAEs/g and from 2.90 ± 0.13 to 4.92 ± 0.21 mg quercetin/g.

A limited number of studies in animals has suggested beneficial effects of dates on plasma lipids [69,70,72,73]. Golden Syrian hamsters fed a high cholesterol diet supplemented with 50% Khalas date pulp (*w*/*w*) for 13 weeks showed significant decreases in plasma cholesterol, triglycerides and LDL-C compared to those consuming the high cholesterol diet alone [69]. Interestingly, the addition of dates to the high cholesterol diet significantly increased liver triglyceride levels compared to the high cholesterol diet alone. Although the mechanisms underlying this liver lipid-loading effect are not clear, fructose, which is present in dates, has been shown to stimulate hepatic triglyceride synthesis when consumed with a hypercaloric diet. Unfortunately, insufficient information was provided regarding the composition of the chow and high cholesterol diets (other than 1% cholesterol added to the chow), or how liver cholesterol and triglyceride concentrations were measured, limiting the interpretation of the reported effects. In another study, hyperlipidemia-induced albino male rats fed either 300 or 600 mg/kg body weight of Aseel date fruit suspension for eight weeks showed significant reductions in triglycerides and very low-density lipoprotein compared to the control group [70]. Curiously, serum levels of cholesterol, LDL-C, HDL- cholesterol and the LDL-HDL ratio were also significantly decreased at the intake of 300 mg/kg but not at 600 mg/kg. Results from the animals receiving date intake at 300 mg/kg approached the same response as the positive control group receiving atorvastatin (2.1 mg/kg). While intriguing, these data must be interpreted with caution, since rodents have a different absorption, distribution, metabolism and excretion (ADME) profile for lipids compared to humans.

A search of English- and Arabic-language databases revealed two human studies on date intake. One is a pilot trial with ten healthy non-smokers consuming 100 g/day of either Medjool or Hallawi dates (equivalent to about seven dates) for four weeks in a crossover design with a four-week washout between groups. A significant 15% reduction in serum triglycerides with Hallawi dates was noted compared to baseline values [72]. However, the baseline triglyceride levels for those who consumed Hallawi dates were considerably higher than that of the Medjool group, although the actual values are difficult to assess since they are only presented by a bar graph. The study also noted that the intake of Hallawi, but not Medjool dates, significantly reduced markers of oxidative stress, as measured by the TBARS assay, the 2,2′-Azobis (2-amidinopropane) hydrochloride-induced serum lipid peroxidation method, and increased serum paraoxonase 1 aryl esterase activity, an enzyme necessary to protect serum lipoproteins from oxidation. The beneficial reductions in oxidative stress after consuming Hallawi, but not Medjool dates, could be related to the total phenolic concentration, which was significantly greater in the Hallawi dates by 20% to 31% [72]. Although the major proportion of the soluble phenolics in both date varieties consisted of phenolic acids, only Hallawi dates contained a significant portion of catechins, which have been reported to exert potent oxidant defense actions. Differences in absorption, metabolism, and bioactivity of the different phenolic compounds in the two date varieties may also help explain the disparate outcomes. Unfortunately, the inclusion and exclusion criteria for participants was poorly defined, and no data were provided regarding whether a return to original baseline values were noted after the washout and crossover period. Due to the high fiber content of dates, which can alter gut microbiome profiles, the influence of a carry-over effect from the intake of one date variety to the next must be considered. Future study designs would ideally include a separate, no-intervention control group as well as a parallel arm design, or ensure that the primary lipid outcome measures along with the gut microbiome milieu at the start of the second intervention period returned to original baseline values.

A second human study assessed the effects of the daily intake of three Khudary dates (tamer stage) for 16 weeks among 100 Bahraini adults with diabetes (39 male and 61 female; treatment group [*n* = 50]) and control group consuming no dates [*n* = 50]). This randomized, controlled, parallel-arm study trial reported a significant improvement in the date group in plasma cholesterol levels of approximately five percent from baseline, along with a trend for reduced LDL-C [23]. However, the results did not reach statistical significance when the treatment group was compared to the control group, suggesting that some of the reduction in cholesterol may have been due simply to participation in the project, independent of date intake. Other limitations include lack of details regarding the actual weight of dates provided, and the overall macronutrient distribution and quantity of the diets.

Taken together, the reports above suggest that dates can improve markers of cardiovascular health, particularly plasma lipid levels, indices of oxidative stress and inflammation, and circulating progenitor cells. These results are primarily from in vitro and animal models, which may be useful as pre-clinical models. Unfortunately, differences in study design, the amount and composition of dates or extracts tested, and lack of details about the control groups fail to provide specificity and limit the ability to draw conclusions about mechanisms and applications to humans. The selection of a control item is important when investigating cardiovascular effects of date products or extracts, because controls can have significant amounts of bioactive compounds that can potentially affect cardiovascular function.

## 5. Potential Physiologic Mechanisms

### 5.1. Polyphenols

Polyphenols are among the most studied categories of dietary phytochemicals in relation to vascular health, and the subgroups of flavanols, anthocyanins and proanthocyanins (PACs) have been of particular interest [11]. Intake of flavanol- and PAC-rich foods and food extracts from strawberries, blueberries, and cocoa have demonstrated improvements in vascular markers that are associated with markers indicative of improved cardiovascular health [74,75,76]. Several molecular mechanisms contribute to the physiological effects of flavanols, including enhancement of vasodilation through the induction of NO [77], free radical quenching (e.g., superoxide and hydrogen peroxide), inhibitory effects on select prooxidants (e.g., nicotinamide adenine dinucleotide phosphate oxidase), and reduction in ET-1 activity [78]. Effects of dietary flavanols on markers of cardiovascular health have been discussed in detail elsewhere [79].

Date cultivar and degree of ripeness are major determinants regarding their polyphenolic composition, as discussed above. The role of date polyphenols has been explored on a number of cardiovascular parameters. Phenolic-acid and flavonol fractions isolated from Amari and Hallawi dates at the tamer stage were examined in vitro for antioxidant and antiatherogenic properties [36]. The two fractions exhibited variable capacities to reduce ferric ions (FRAP assay), scavenge radicals and inhibit LDL-C oxidation via TBARS and lipid peroxide assays, with the flavonol fractions showing the strongest effects. Only the flavonol fractions stimulated cholesterol removal from macrophages. The yields of isolated fractions in terms of μmol GAE per g fruit were considerably larger for Amari dates by approximately 10- and 3.5-fold for phenolic acids (Amari: 4.3 μmol GAE/g; Hallawi: 0.38 μmol GAE/g) and flavonols (Amari: 1.4 μmol GAE/g; Hallawi: 0.4 μmol GAE/g), respectively. The two isolated fractions contained ferulic acid as a major component and comparably small amounts of coumaric acid, but differed considerably in the composition of their complementary set of phenolic acids. Amari dates contained primarily caffeic acid derivatives, whereas the Hallawi variety contained mostly a salicylic acid derivative. Seven prominent peaks of flavonols were evident. Based on an authentic standard library, all seven flavonols were tentatively classified as kaempferol derivatives. The two isolated fractions of date flavonols differed considerably in composition. In addition to one prominent flavonol peak shared by the two fractions, Amari consisted of significant amounts of five other flavonols, whereas Hallawi contained a single unique flavonol as the major component. The results demonstrated strong structure-activity relationships for date polyphenols and identified date flavonols as potential antiatherogenic bioactives. 

Polyphenols derived from date syrup at concentrations of 60 and 600 μg/mL, predominantly from cinnamic acid (73.8 mg/100 g) and catechin (42.7 mg/100 g) derivatives, were found to significantly attenuate IL-6, IL-8 and vascular endothelial growth factor (VEGF) in human vascular endothelial cells (HECVs) [67]. These observations corresponded to a significant reduction of both cyclooxygenase-2 and VEGF induced by TNF-α at both the protein and gene expression levels in the assessment of inflammatory-associated angiogenesis in HECVs.

Many polyphenols found in dates have been studied as isolated compounds in in vitro and ex vivo systems with respect to their effects on markers of vascular function. Protocatechuic acid, a metabolite of the anthocyanin cyanidin-3-glucoside (C3G), and its phase II metabolites were effective in modulating the production of the key inflammatory mediators IL-6 and vascular cell adhesion molecule-1 (VCAM-1) at dietary-relevant concentrations as low as 100 nmol L^−1^, with maximum reduction observed for the sulfate conjugates in human umbilical vein endothelial cells (HUVECs) stimulated with either oxidized LDL or a cluster of differentiation CD40L [80]. In the same study, C3G and its metabolites reduced IL-6 production in CD40L-stimulated cells, whereas both C3G and its metabolite, ferulic acid, reduced VCAM-1 production. Anthocyanins and ferulic acid have also been found to significantly reduce monocyte adhesion to HUVECs under physiologically relevant conditions, an important step in reducing atherosclerosis development [81].

In human intestinal cells in vitro, the addition of a freeze-dried date extract from California-grown dates (Deglet Noor and Medjool varieties) with a total proanthocyanidin (PAC) content of 13% (dry weight basis; 131.3 mg PACs/g of date palm extract) was demonstrated to act as a potent co-agonist ligand for the farnesoid x receptor (FXR), a nuclear structure important for maintaining triglyceride and cholesterol homeostasis [68]. This study provides a potential mechanism by which dates may exert a hypotriglyceridemic effect, as observed in the human study noted above [72]. The tea catechin, epigallocatechin-3-gallate (EGCG), has also been shown to modulate FXR in a tissue- and gene-specific manner [82]. Further studies are warranted with dates and their extracts using both wild-type and FXR knockout mouse models.

While the above in vitro work is promising, data from dietary interventions that specifically examine the association between circulating date polyphenols or phenolic metabolites with physiological effects have yet to be reported. Additionally, clinical studies are needed, since in vitro and animal studies, while potentially relevant as preclinical models, do not directly assess outcomes such as the vascular effects in humans.

Clinical and mechanistic data on the biological effects of polyphenols derived from dates are limited (Table 1). Such evidence is crucial for agriculture, health professionals and consumers, particularly as the concept of personalized nutrition grows more popular. In addition to randomized clinical trials on the fruit in general, more data is needed to identify which polyphenols are the most vasculoprotective and then determine how best to cultivate, harvest and process the fruit for maximum bioactivity. The efficacy of date consumption also needs further interrogation in various at-risk groups such as those with lipid disorders, hypertension, obesity or diabetes. Since all fruits are not equal in composition, identification of unique bioactive compounds or fractions in dates would help define benefits that may not be obtained from other fruits or plant-based foods.

### 5.2. Other Vasculoprotective Nutrients in Dates

Beyond polyphenols, dates contain cardiovascular-protective nutrients including potassium, magnesium, folate, selenium, fiber, and vitamin C (Table 2). Most date varieties are rich in potassium and low in sodium, both of which are important dietary factors that help maintain blood pressure in the normal range [83,84].

Dates contain folic acid and vitamin C. Folic acid is required to metabolize homocysteine to methionine [86]. Elevated levels of serum homocysteine have been associated with increased risk for CVD [87,88]. Although the mechanisms by which increased homocysteine promotes CVD are incompletely defined, suggested alterations include impaired vascular tone due to decreases in NO bioavailability and increases in ET-1, promotion of damaging ROS, and endothelial inflammation and the activation of the coagulation cascade [89]. The vitamin C in dates, while modest in amount compared to most citrus fruits, can nonetheless help to scavenge free radicals via enzymatic and non-enzymatic activities, and help protect lipoproteins from oxidative damage [90,91]. In addition, vitamin C can improve measures such as arterial stiffness and endothelial function [92,93], and low serum concentrations of vitamin C have been linked to increased CVD risk (i.e., incident cases of heart failure) [94] and mortality [95].

Some of the cardioprotective effects of dates have been ascribed to dietary fibers, which have a well-established lipid-lowering effect [96,97]. Serum triacylglycerol, total cholesterol, and LDL-C levels were significantly lowered in rats given 100 g/kg of date dietary fibers [98]. Most of the fibers in dates are insoluble. These fibers can bind to cholesterol and triacylglycerols in the intestine and facilitate their excretion, which helps lower circulating cholesterol levels [99,100]. As a result, less lipoprotein is also susceptible to oxidation, thus reducing the impact on atherogenesis [97]. Further, fiber-rich foods can promote production of beneficial commensal bacteria while limiting the growth of known opportunistic pathogens [101]. A high-fiber diet was reported to increase acetate-producing microbiota, lower blood pressure and decrease cardiac hypertrophy and fibrosis in hypertensive mice [102]. Bacterial fermentation of prebiotic soluble fiber generates short chain fatty acids, which are thought to exert several beneficial effects including differentiation of immune regulatory T cells, and decreasing the expression and activation of peroxisome proliferator-activated receptor-γ (PPAR-γ) [103,104]. Downregulation of PPAR-γ activates a mitochondrial uncoupling protein 2 and an AMP-activated protein kinase network, shifting metabolism in adipose and liver tissue from lipogenesis to fatty acid oxidation [104]. Conversely, activation of PPAR-γ has been shown to have anti-inflammatory effects, promote the expression of genes for fatty acid oxidation, and decrease lipotoxicity in macrophages [105].

The interaction of date fibers and polyphenols may also impact vascular function. The gut microbiota are critical for enhanced bioavailability and activity of ingested polyphenols, as most parent compounds are not well absorbed in the small intestine [106]. Following the ingestion of polyphenols, typically in their glycosylated forms, bacteria in the gastrointestinal tract metabolize these molecules to low-molecular-weight phenolic compounds [107] that are then absorbed by intestinal epithelial cells [108]. Polyphenols have been shown to undergo a variety of enzymatic processes by bacterial populations in the gastrointestinal tract, including the hydrolysis of glycosylated flavonoids, acylation of flavanol-3-ols and esterification of hydroxycinnamic acids [109]. A detailed description of these mechanisms can be found elsewhere [11,110].

## 6. Future Considerations

While the health promoting cardiovascular benefits of a number of fruits, nuts and berries rich in select polyphenols (e.g., walnuts, strawberries, apples) have been characterized through animal and human studies [76,111,112,113,114], no such data exists for dates to our knowledge. Given the polyphenol and fiber content of dates, vascular function and gut microbiome studies would be useful. Vascular function is commonly assessed by two noninvasive techniques: flow-mediated dilation (FMD) of the brachial artery and peripheral arterial tonometry (PAT) in the fingertip [115,116]. Both methods have demonstrated prognostic value for the assessment of cardiovascular risk factor burden [117,118,119]. High dietary intakes of select polyphenols, such as the flavanols and PACs found in berries, tea, red grapes, and cocoa have been reported to significantly improve FMD and PAT in various population groups [74,120,121,122]. Importantly, no data exists with respect to the impact of date products and their polyphenols on vascular dysfunction via measurement of FMD and PAT.

An inherent challenge with most nutrition studies is the identification of suitable controls. This is particularly difficult when examining the potential health effects of whole foods that contain a multitude of compounds that are bioactive either separately or through their interaction with other constituents in the food matrix. One model for testing foods or extracts is to use a control product that is closely matched in calories, macro- and micro-nutrients, taste, and color, but devoid of the test fraction or compound. This model has been used successfully in studies that assess the effects of a dietary strawberry powder [113] and a flavanol-rich cocoa drink [123]. Another model is to employ a no-intervention control group, although operationally, this may skew the results since a number of those assigned to the control group may withdraw from the study prior to its completion, and those remaining may not fully represent the population initially enrolled.

Future human research on dates must select the study population carefully, and focus mainly on groups at risk for CVD. Accordingly, hormonal status, age and sex are factors that can produce significant interindividual variability in cardiometabolic responses to phenolic compounds and must be considered. Factors such as microbial metabolism and genetic polymorphisms may be other contributors to outcome variability [124].

Recent attention has also emphasized the challenge of reproducibility and accuracy in human nutrition research [125]. As noted above, more complete compositional profiles of dates are needed rather than simply recognizing the total amount of GAEs, a gross index of flavonoid content. A more detailed characterization of products, reagents, and model systems used, as well as better rigor and reporting of experimental designs, protocols, and data analysis, will help achieve this goal. Worthy of note, many of these elements were limiting factors in the in vitro and in vivo animal reports discussed above.

### Safety Concerns

While dates have positive biological effects, concerns have been raised about their potential concentrations of heavy metals [126,127]. Exposure to heavy metals can result in cardiovascular diseases, encephalopathy, renal dysfunction, dementia, and certain cancers [128,129]. A recent study of seven date varieties collected from different locations in Saudi Arabia noted that aluminum, chromium, and antimony were within a safe range based on the maximum allowable levels set by the World Health Organization, while arsenic, lead, and cadmium exceeded the upper limit in some of the date cultivars [126]. Numerous environmental factors can increase concentrations of heavy metals such as mining, fertilizer applications and industrial emissions, as well as naturally occurring amounts normally found in some soils [129].

## 7. Conclusions

Emerging data supports the notion that intake of dates can have beneficial effects on markers of vascular health. The majority of effects have been observed in response to the whole fruit or extracts in animal models, providing a strong rationale to conduct randomized clinical trials and epidemiological investigations. Intake of date products or their polyphenol fractions seem to favorably modulate plasma lipid levels, indices of oxidative stress and inflammation, all of which are responses associated with improved cardiovascular health. In addition to measuring changes in cholesterol or markers of oxidant defense, assessment of functional markers would provide useful information. Trial designs that capture the relationship between circulating metabolites from dates or date-derived polyphenols and physiologic responses would also be helpful. Since current recommendations emphasize dietary patterns that are abundant in plant foods, dates may be an excellent food to help meet these goals.

## Figures and Tables

**Table 1:** Intakes of date palm fruit products and surrogate cardiovascular markers ^a^.

Product	Model	Quantity	Duration	Response
Dates	In vitro (colorimetric assay)	No data available	-	↓ ACE activity [60]
Dates	In vitro	100 g dry weight	-	↑ TEAC, ABTS^•+^, FRAP [65]
Date phenolic acid and flavonol fractions	In vitro	Amari PhA: 4.27 μmol GAE/g Hallawi PhA: 0.38 μmol GAE/g Amari Fl: 1.37 μmol GAE/g Hallawi Fl: 0.43 μmol GAE/g	-	All PhA and Fl fractions: ↓ Ferric ions, scavenge radicals, LDL oxidation via TBARS and lipid peroxide assays Only Fl fractions: ↑ Cholesterol removal from macrophages [36]
Date syrup derived-polyphenols	In vitro (HECV cells)	60 and 600 μg/mL	-	↓ IL-6, IL-8, VEGF ↓ COX-2 & VEGF induced by TNF-α at protein level and gene expression [67]
Date extract	In vitro (Human intestinal cells; Caco-2 cells)	100 mg	-	Acts as a potent co-agonist ligand for FXR Regulates FXR-target gene expression in Caco-2 cells [68]
Dates	Male golden Syrian hamsters	Date pulp mixed with chow powder (1:1, *w*/*w*)	13 weeks + HCD	↓ Serum cholesterol, TG, LDL [69]
Date fruit suspension	Hyperlipidemic rats	300 and 600 mg/kg	8 weeks + HFHSM	300 mg/kg: ↓ Serum cholesterol, TG, LDL, VLDL, C-HDL, LDL-HDL 600 mg/kg: ↓ TG, VLDL [70]
Date palm seed	CCl4-induced Hepatotoxicity in Rats	1.0 g/kg	4 weeks	↓ AST, ALT, ALP, TBARS, NO, liver lesions ↑ SOD, GST [61]
Date palm seed	Streptozotocin-induced diabetic rats	1 g/kg/day	4 weeks	↓ ALT, AST, TBARS, NO ↑ SOD, GST, CAT [62]
Date extract	Ex vivo: DCFH-toxicated cardiomyoblast cells (H9C2) In vivo: IPS-injured Wistar rat heart tissues	Ex vivo: 250 µg/mL In vivo: 250 and 500 mg/kg body weight	-	Ex vivo: Attenuated cytotoxicity and enhanced H9C2 proliferation (40%) In vivo: ↑ CAT, SOD, NO, Bcl2 ↓ IL-6, IL-10, TNF-α, MDA, caspase-3 and Bax [63]
Date pollen extract	Cadmium-induced testicular dysfunction and oxidative stress in rats	40 mg kg^−1^	56 days	↓ MDA ↑ Glutathione [64]
Dates	Doxorubicin-associated cardiotoxicity in rats	1.4 g/kg	Single dose	↑ Rate of rise in left ventricular pressure (d*p*/d*t*_max_) and (d*p*/d*t*_min_), glutathione ↓ QT interval, JT interval, and *T*_peak_-*T*_end_ interval [66]
Date extract	Induced myocardial infarction in rodents	200 and 400 mg/kg body weight	28 days	↑ GSH, SOD, CAT ↓ TBARS, troponin-T, LDH, CK, and AST ↑ CD34 and CD133 positive progenitor cells Improved histopathological indices of MI [71]
Dates	Healthy participants (*n* = 10)	100 g/day	4 weeks	↓TG, TBARS, and AAPH-induced serum lipid peroxidation ↑ PON1 aryl esterase activity [72]
Dates	Diabetic participants (*n* = 100)	3 dates/day	16 weeks	↓ Total cholesterol Suggestive ↓ LDL [23]

DPF: dried palm fruit; CCl4: carbon tetrachloride; DCFH: dichlorodihydrofluorescein; ISO: isoproterenol; HECV: human vascular endothelial cells; PhA: phenolic acids; Fl: flavonols; GAE: gallic acid equivalent; HCD: high-cholesterol diet; HFD: high-fat diet; HFHSM: high fat high sugar diet; ACE: angiotensin converting enzyme; TBARS: thiobarbituric acid reactive substances; AAPH: 2,2′-Azobis (2-amidinopropane) hydrochloride; PON1: Paraoxonase 1; MDA: malondialdehyde; C: cholesterol; TG: triglycerides; HDL: high-density lipoprotein; LDL: low-density lipoprotein; VLDL: very low-density lipoprotein; AST: aspartate transaminase; ALT: Alanine aminotransferase; ALP: Alkaline phosphatase; ROS: reactive oxygen species; NO: nitric oxide; GSH: glutathione; SOD: superoxide dismutase; GST: glutathione S-transferase; CAT: catalase; IL-6: interleukin 6; IL-10: interleukin 10; TNF-α; tumor necrosis factor-alpha; Bcl2: B-cell lymphoma 2; Bax: bcl2-associated X protein; LDH: lactate dehydrogenase; CPK: creatine phosphokinase; CK: creatinine kinase; CD: cluster of differentiation; VEGF: vascular endothelial growth factor; COX-2: cyclooxygenase-2; FXR: farnesoid x receptor. ^a^ Includes cell, animal, and human clinical studies of known physiologically relevant measures related to cardiovascular health.

**Table 2:** Reported micronutrient and fiber content of select date fruit varieties per 100 g.

Nutrient	Content/100 g	% US RDA for Adults
Dietary fiber (g)	4.7–7.9 [39,72,85]	No RDA; AI instead Men 19–50 years:12.4–20.8 Men over 50 years: 15.7–26.3 Women 19–50 years:18.8–31.6 Women over 50 years: 22.4–37.6
Vitamin C (mg)	3.9 [26]	5.2
Calcium (mg)	50–71 [26,72]	4.2–5.9
Iron (mg)	0.3–6.03 [26,72]	Men: 3.75–75.38 Women: 3–33.5
Potassium (mg)	525–864 [26,72]	No RDA; AI instead Men: 15.4–25.4 Women: 20.2–33.2
Folate (mcg)	15–19 [24]	3.8–4.8
Magnesium (mg)	50–64 [26,72]	Men: 12–15 Women: 16–20
Selenium (mcg)	340 [42]	618

## Data Availability

Not applicable.
